# Magnitude of the Quality Assurance, Quality Control, and Testing in the Shiraz Cohort Heart Study

**DOI:** 10.1155/2020/8179795

**Published:** 2020-08-11

**Authors:** Nader Parsa, Mohammad Javad Zibaeenezhad, Maurizio Trevisan, Ali Karimi Akhormeh, Mehrab Sayadi

**Affiliations:** ^1^Cardiovascular Research Center, Shiraz University of Medical Sciences, Shiraz, IR, Iran; ^2^City College of New York Provost & Senior Vice President for Academic Affairs, Dean of Medical School, New York, USA; ^3^College of Health Sciences Vin University Hanoi, Vietnam

## Abstract

To determine the conclusive integrity in the Shiraz Cohort Heart Study (SCHS) project, management began quality assurance (QA) and quality control (QC) of the collected data throughout the study end-points. The QA is a focused process that prevents and detects data collection errors and verification of intended requirements in the SCHS. The QC is a subset of QA intended to capture errors in processing data through testing and preventive processes to identify problems, defects, or intended requirements. SCHS involved 10,000 males and females aged 40-70 over a 10-year follow-up period with cardiovascular diseases (CVDs) in the city of Shiraz, Iran. The study measured events and access to preventive care in Shiraz city. The SCHS identified unique barriers to select national study models in developing standardized measures related to variations in ethnicity, religion, cross-cultural considerations, and others. A suggested response to this problem was to develop a mechanism to standardize elements of the questionnaire, study design, and method of administration. This action was based on the geographically normal distribution of the Family Physician Health and Medical Services in Shiraz. Important QA and QC decisions were developed and adopted in the construction of the SCHS and follow-up to ensure conclusive integrity.

## 1. Introduction

Since the development and refinement of QA, QC, and testing tools in clinical research, the planning and conduction of large studies improved greatly. A substantial literature on QA and QC has appeared in the framework of cohort studies and clinical trials. There has been an emphasis on data quality improvement through standardization of research protocols and education of personnel involved in study and data managing systems [1–3]. Moreover, QA and QC tools were developed for the planning, execution, and effective analysis of epidemiological studies [4]. Description of QA and QC procedures are largely in the context of a study Greenberg et al. [5], the Hypertension Primary Prevention Trial [6], or the Optic Neuritis Treatment Trial [7, 8], with some guidelines given within several articles [9, 10]. There are not many found in the literature in the framework of nonclinical trial studies, even though some study detailed approaches [4, 11, 12] and general QA and QC guidelines have been addressed [13–15]. There were fewer longitudinal research studies found that exclusively describe QA and QC in their projects to achieve international quality standardization [5, 10, 16, 17].

The present evaluation aimed to describe and apply QA-QC operations related to the SCHS in the sequence within which they were developed.

### 1.1. Rationale for QA, QC, and Testing of the SCHS

QA was applied as a preventive process prior to data collection, while QC is a corrective function completed at the beginning and through the end of the data collection process in order to identify and correct errors or discrepancies in the data that are encountered during the entire SCHS.

Key QA and QC decisions were adopted in the construction of this cohort, its follow-up, and integrity of the conclusions drawn of the collected data. Testing was accomplished as a subset of QC as preventive operations that ensured the identification of problems, errors, and defects in software or predetermined requirements. Verification of study ensured that the software system met all types of functionality and subsequently ensured that the functionalities met the intended behavior of validation. As a result, verification, validation, investigation, inspection, and audit were strictly applied for minimizing and eradicating all potential sources of type-I, type-II errors, discrepancies of transcription at data entry and data manipulation for analysis to prevent systemic errors which could lead to an incorrect report of data relations, and poor quality data which could decrease the power in the study.

## 2. Method and Material

The study aimed to comprehensively follow-up 10,000 males and females in an exclusive-center, based on interviews and tests of varied complexities and diverse ethnicities over a 10-year period with greatly developed QA, QC, and testing tools among Family Physician Health and Medical Services, private clinics, or organizations with geographically normal distributions of the Shiraz metropolitan city.

The magnitude of the SCHS is as a unique metropolitan city cohort study accomplished by careful selection of research instruments. The training, accreditation with individual staff certifications, pretesting, pilot study, and preparation of operation manuals for the methods were all done within the SCHS study facilities.

Evaluation of the baseline data collection followed the approved questionnaires. Follow-up to the questionnaires were communication with the many subgroup populations to standardize the wording of the questions. This gains better understanding of the characteristics within the subgroups and increased understanding by the interviewers. In addition, the questionnaires should be complete and clear as well as the person giving the interview, and the mechanical instruments and technical measurements need to be accurate.

Routinely, QA, QC, and testing were repeated during the beginning, throughout, and after the gathering phase of data with the intent to improve the design and final performance on completion of the operation.

Following the study design and methods, administration of the SCHS geographically normal distributions model combines multiple Family Physician Health and Medical Services base locations into an exclusive single-center cohort study and laboratory test results for data collection that automatically prevent any further QA and QC implementation. A preferred single-center model compared to multiple-center model is more beneficial and exclusively subject to maximum and precisely directed supervision for QA, QC, and testing related to long-term; data collection, clinical laboratory test results, storage biobank samples, and other issues in terms of changes over time; in addition, relationships between QC supervisor, site personnel and to the management of performance at individual sites. In this study, QA, QC, and testing intermittent operations were carried out comprising checkup including the site technicians, test-retest studies, and the monitoring of data through a system of cross visits and supervisions. The dependability of the information was estimated from data that testified the achievement of quality goals. The SCHS QA, QC, and testing systems were according to international experiences, while necessary adjustments were performed by the Steering Committee and its Advisory Committees, on the principles determined by the QA and QC Committee.

**Figure 1:**
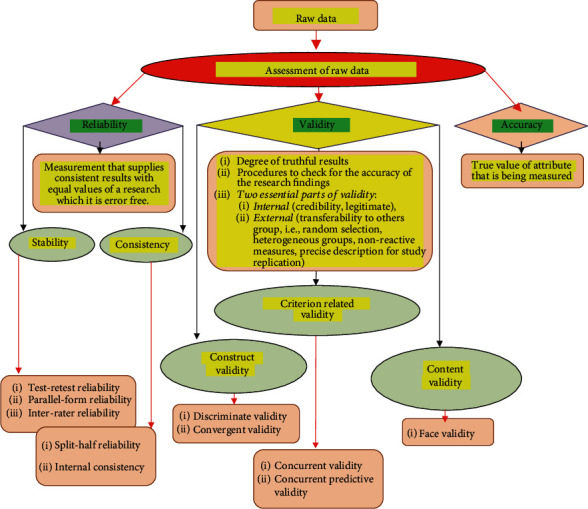
Assessment and Magnitude of Reliability, Validity and Accuracy related to Raw Data Quality

In summary, methodological assessment and magnitude of reliability, validity, and accuracy related to raw data quality are indicated in Figure 1.

## 3. Results of QA and QC Components Implementation

### 3.1. Result of QA Implementation

As a result, the major implementation component of the QA process was related to protocol development and the creation of operational documents for the SCHS. The operations manual, with clear and detailed descriptions, constituted the study's navigation guidance of all of the activities and was a task vital in the planning for the research team and investigators.

Therefore, objectives and research design, detailed attributes of the population to be studied, sample size, and selection of the instruments to obtain relevant information, including logistic, functional, and financial standpoint by the SCHS Steering and Advisory Committees were developed. The study design of the data collection instruments contained content, format, and step-by-step instructions for finalizing the instrument. The limitations of multiple types of instruments were studied during the development phase so that problems were identified and lessened to the greatest extent possible.

The Operations Committee addressed questions that may arise about protocol implementation to safeguard that the protocols are followed and to address difficulties that may arise. The QA and QC committees monitor the actual data quality and address minor problems for corrections by periodic site cross visits.

We tested volunteers that were like the planned cohort with self-administered questionnaires to identify any problems in the questions prior to the initiation of the actual study. We were looking for any obstacles in response to interviewers that were different to the respondents due to differences in age, gender, or ethnicity. Interviewers were also directed in how to respond to questions by the participants to further clarify the response.

All perspectives of the SCHS protocol were documented in a manual of operations [17]. Therefore, when the operations manual was created, there was a process to review and correct any section that seemed ambiguous or subject to misinterpretation. Furthermore, SCHS methods were designed to make sure the data produced were accurate, reliable, and valid according to periodic recalibration of equipment and replacement of defective equipment, and do not reflect bias that may arise in subgroups of Family Physician clinics or organization and over time being studied. Also, various equipment used to collect and measure data was standardized and identical throughout the cohort that minimized variabilities.

Consequently, SCHS committee has developed methods for reviewing and updating the protocol and to communicate changes to all study personnel as needed. We conclude the skills and professional dedication individuals had direct bearing on the final quality of data in the study. Therefore, this committee developed procedures to obtain and maintain performance certification of study procedures and processes for monitoring those requirements throughout the course of the study. Practically pivotal testing was conducted for all study personnel to ascertain reliability of self-reported data of self-administered questionnaires to identify any problematic questions before data collection and data entry. For more ascertainment in some cases, we double verified to capture and correct errors of data entry from the original entry.

### 3.2. Result of QC Implementation

As a resultant, the leading accomplishment constituent property of the QC was to identify and correct the causes of either bias or unwarranted disorder in the data sets in any stage of study. The complexity related to QC of SCHS activities was divided into initial, during, and after stages of those issues relevant to the field and reading centers or laboratory. The following processes were necessary for QC procedures of this study before discussing the different stages of management throughout the collection of data.

Moreover, staff incentives for appropriate QC assessments and data collection in this study were encouraged to obtain top quality work. This was achieved in reminding staff that they were an integral part of the larger study to encourage interest in the quality of their work. Periodic QC staff engagement surveys and reviews of work done by staff were sent to the SCHS center to identify how well the site was doing in meeting recruitment goals as well as the total recruited toward the target goals that included gender and ethnicity to make sure participants met the study eligibility criteria.

Experience identified that the QC director should not be the person observing and monitoring the interview as this may negatively impact the technique of the interviewer and the participant's responses. Therefore, the SCHS monitoring key personnel were forbidden to approach these activities.

Literally, in data cleanup of discrepancy issues, the coprincipal investigator routinely analyzes related problems and detecting errors such as extreme or inconsistent values for accuracy purposes. In this study, multiple measures for some variable such as blood pressures due to variations were taken at one point in time to identify possible data errors and to calculate finer measures of data accuracy.

In order to address potential problems that could adversely affect the quality of the study, such as staff turnover and technician drift, staff were required to be certified at the SCHS center along with requirements for a minimum number of procedures to be performed (weekly or monthly) to maintain their certification and thus minimize drift. Furthermore, field center QC activities included efficient training updates by Shiraz Cardiovascular Research Center scientists and recertification throughout the course of the study that ensured minimal QC problems and a major impact on data quality and refreshing protocol.

It was important that equipment be maintained and calibrated regularly in the QC study research center, for all aspects of field study (such as scales calibration and freezers temperature) to minimize any measurement bias or error related to the equipment.

The QC protocols included directives for the cleanliness of the work environment that included sanitizing of equipment (cuffs for measuring blood pressure) and quantitative control of the materials to perform tests and measurements (electrodes, ECG paper, gauze, gloves, alcohol 70%, conductive gel, and etc.). In addition, the room's temperature for blood sample and measurement of blood pressure was monitored throughout the day maintained between 20°C and 24°C.

The final step of the initial QC in this study was transferring blood samples to laboratory for processing (serum, DNA extraction, Buffy Coat) and biobank storage at the -86°C freezer in the central laboratory of the Research Tower for subsequent analytical purposes.

During the collection of the data and before entering the data into the tracking and management systems, we believed there would be some inconsistent participant response over time. Thus, data are collected by well-trained interviewers, as some data had limitations or possible suspension (such as a few proportions of participants reporting one time that they are current smokers and another time they were never smokers). The possible QC solutions were to again contact the participants who gave inconsistent responses and seek clarification. This process helped to verify the data by filling in the missing and inconsistent values. Therefore, in this regard, the quality control of appropriate answers as a repeated measure procedure was conducted for more clarification and consistency of collected data.

## 4. Discussions of QA and QC Components Implementation

### 4.1. Discussion of QA Implementation

In general, for QA accomplishments to achieve more relevant practical provisions to each situation, pretest and testing of all instruments and procedures were implemented. These performances were pretested and tested in the research context by way of a pilot study in which the entire protocol is completed on volunteers who are demographically like the anticipated SCHS cohort. The pretesting and then testing of the SCHS instruments and procedures, before we included them in the operational manual for the project, were essential to begin the training of the research team.

The unique authenticity of the SCHS study required validation of the instrument as it relates to various ethnicities, religion, cross-cultural considerations, and other design features before it was adopted by the real study.

When the SCHS protocol was developed and documented, training and issuing certification for study personnel, according to the specificities of each procedure, were implemented. Central and identical training was pivotal as it had a direct impact on the interviewers or technicians to perceive the value and consistency of the data. The rationales of training and certification activities resulted in standardization which crucially reduced costs over time. At the end of the training process or when later research team licenses were issued.

Practical procedural lab training included interviewing, blood sampling, and processing (serum, extracted DNA, Buffy Coat) as well as freezing and transporting to the long-term biobank storage (-86°C).

Eventually, while training of the study team was completed, the pilot study was serially conducted with increasing complexity. All validity and reliability performances related to the QA process of study were applied. The SCHS pilot study involved all features of the protocol including interviews, computed variables, entry and transmission of the data to a coordination center, and dispatching samples to the reading center or laboratories.

### 4.2. Discussion of QC Implementation

Since this study is interested in measuring real change in outcome variables over time, QC procedures assessed and minimized irrelevant variability in these exposure measures that were vital. It should be noted that to separate random biologic variability, measurement error, and true change was difficult. However, it was essential to obtain good estimates of biologic variability and measurement error so that true change could be measured. For this purpose, we approached the available procedure. These proceedings were calibration set at some point early in the study at the reading center or laboratory in a blinded manner at regular intervals with the results tabulated at the SCHS coordination center. This means identified comprehensive drift over a period of time or the introduction of bias into the data.

Therefore, data were carefully reread by the assigned readers in a blinded fashion designed substudy carried out to assess and give estimates of the interreader and intrareader variability.

When estimations of interreader and intrareader variability were presented, the study allowed the estimation of additional characteristics of variability such as field center technician and biologic variability combined. The combination of a reader and technician effect affords an overall measurement error. Therefore, this measurement error could result in any type-I or type-II errors.

In the SCHS, when a continuous data outcome variable was measured with error, we presumed a type-I error may have occurred. Thus, to best explain and clarify the concern, we applied a regression analysis to look at the association between the outcome variable and a set of exposure variables and adjust for the baseline value of the outcome variable. This result could identify a relationship between observed changes in the outcome and exposure variables even when there was no relationship no association existed between the variables and the real change in the outcome variable that was supported by previous research [18].

In this study, elevated levels of random measurement were thought prone to type-II error, and this may conceal a true association between outcome variable and a set of exposure variables.

In the SCHS study, QC analyses were pivotal to data processed at a reading center or laboratory over a frame of time. Quality problems that led to a type-II error could be encountered and may involve falsification by staff at the QC reading center and laboratory to falsely show improved efficiency in these centers. When falsification was suspected, the techniques of set calibration or longitudinal plots were applied. When falsification was identified, the reader was retrained until performing at an acceptable level before data processed by the technician would be analyzed to see if a statistical correction were achievable or dismissed.

Also, to achieve minimum data loss due to mishandling, mislabeling, or other obstacles for laboratory data, the SCHS well organized the reading center to monitor processing and in cooperation with the coordinating center made sure that enough systems were available to track data between the SCHS field, reading and coordination centers.

Ethical criteria defined by the study implied the adoption of certain processes to pledge the confidentiality of information, such as registering only the recruitment number on the forms and questionnaires [19]. The list with names and recruitment numbers was the responsibility of the director, and it was disclosed only in some situations, like to relay test results. The research team signed a data confidentiality agreement.

Practically, initial data collection started with the pilot group with gradual increase planned according to the experience of the research team linked to the protocol, flowchart of tests, and interviews. This enabled a more thorough QC that led to the creation of field diaries, which were revised daily and discussed in meetings weekly with the QC team. These tasks were carried out and shared with the QC director of the study center directly or by electronic mail, discussion group, by telephone, or over the internet. At this stage doubts, uncertainty and falsification of records were identified specific problems reviewed, annotated, and commentated to the responsible people for final correction.

After the initial difficulties were overcome, the detailed checklists that were adopted at the initial stage of data collection by the SCHS steering committee were revised and simplified to be used for the final study. Periodically, the interviews of a certain week were recorded, and randomly, some were evaluated by a coprincipal. At this step, emphasis was given to the interview's fluency, the correct completion of the answers, and suitability in the participant communication.

When data collection and entry occurred in the system, the correct marking of the answers of the interview (missing, unknown, blank answers, skip errors, or other inconsistencies) was made by the system. Random repetition of measurements by the same person or by another was used for some tests and questionnaires. At this point, consideration was taken so that the measurements by the same interviewer or technician could not remember the previous results, and that, the test and retest results were independent. This procedure also used for the reliability of laboratory and echocardiography and was not more than 15-20 minutes. For the degree of agreement related to test-retest of accuracy, reliability, and validity, Cohen's Kappa statistical coefficients were implemented [20].

A final aspect of field center data quality must be considered in the SCHS related to inconsistent participant response over time. Thus, data were correctly collected by interviewer staff, though the limitations of reported data in advanced are well-known. As an example, a few participants reported initially that they were current smokers and then later that they were never smokers. Thus, for this purpose and clarification of suspicious inconsistent data, a double-blind retest was conducted at each visit for some data. Findings of any artificial or actual triggers such as unknown, missing, incomplete, and lack of homogeneity of data for each follow-up event, we applied quality control measures to find problems by the random double-blind test and retest, for any inconsistencies in data collection. We performed daily and weekly quality assurance, quality control, and testing before data entry, to remove any risk of influencing environmental, societal, or emotional factors. However, if the coprinciple of the study assumes there were problems, that person directly reevaluates and retests with another reviewer. To enhance quality assurance and quality control and certainty, double-blind checking by Kappa statistical coefficients agreement data is applied to locate any inconsistent data. The quality control protocol should recognize that such inconsistencies will occur and have a method in place for handling them and clean up data. Possible solutions are to recontact participants with inconsistent responses and ask for clarification, to set inconsistent responses to missing values, or, if possible, to use an independent source (such as laboratory results and other related collected data) to verify the data and report to the Advisory Committees. If data was still incomplete and not able to correct after contacting participants and an in-depth revision and assessment of collected data before data entry, the coprincipal of the SCHS and QA and QC teams were approached for the solutions.

Consequently, in this study, after in-depth assessment and revision of collected data and data entry by the coprincipal of the SCHS QA, QC teams approximately 2% of the files had minor inconsistency according to a previously defined script. This finding was produced to meet the study's requirement goals, and data incorporated into the system were also generated for exploitation. Moreover, the magnitude of the QA and QC processes in this study required a considerable review of study resources and should not be underestimated.

## 5. In Conclusion

We achieved a well-designed SCHS study with in-depth QA and QC proceedings by the coprincipal of the study and dedication by the research team to the process, accuracy, reliability, validity, and integrity of conclusions can be established, and the acquired experience would be useful to other cohort studies.

## Data Availability

At this point, “not applicable” in current manuscript.
